# Viruses, parkinsonism and Parkinson’s disease: the past, present and future

**DOI:** 10.1007/s00702-022-02536-y

**Published:** 2022-08-29

**Authors:** Valentina Leta, Daniele Urso, Lucia Batzu, Yue Hui Lau, Donna Mathew, Iro Boura, Vanessa Raeder, Cristian Falup-Pecurariu, Daniel van Wamelen, K. Ray Chaudhuri

**Affiliations:** 1 Department of Basic and Clinical Neurosciences, Institute of Psychiatry, Psychology & Neuroscience, King’s College London, Cutcombe Road, London, SE5 9RT UK; 2 Parkinson’s Foundation Centre of Excellence, King’s College Hospital, London, SE5 9RS UK; 3 Department of Clinical Research in Neurology, Center for Neurodegenerative Diseases and the Aging Brain, University of Bari ‘Aldo Moro’, “Pia Fondazione Cardinale G. Panico”, Tricase, Lecce Italy; 4 Department of Neurology, Donders Institute for Brain, Cognition and Behaviour, Radboud University Medical Centre, Nijmegen, The Netherlands; 5 School of Medicine, University of Crete, Heraklion, Crete Greece; 6 Department of Neurology, University Hospital of Heraklion, Heraklion, Crete Greece; 7 Department of Neurology, Technical University Dresden, Dresden, Germany; 8 County Clinic Hospital, Faculty of Medicine, Transilvania University, Brașov, Romania

**Keywords:** Virus, Parkinsonism, Parkinson's disease, Infection, COVID-19, SARS-CoV-2

## Abstract

Parkinsonism secondary to viral infections is not an uncommon occurrence and has been brought under the spotlight with the spread of severe acute respiratory syndrome coronavirus 2 (SARS-CoV-2) infection. A variety of viruses have been described with a potential of inducing or contributing to the occurrence of parkinsonism and Parkinson’s disease (PD), although the relationship between the two remains a matter of debate originating with the description of encephalitis lethargica in the aftermath of the Spanish flu in 1918. While some viral infections have been linked to an increased risk for the development of PD, others seem to have a causal link with the occurrence of parkinsonism. Here, we review the currently available evidence on viral-induced parkinsonism with a focus on potential pathophysiological mechanisms and clinical features. We also review the evidence on viral infections as a risk factor for developing PD and the link between SARS-CoV-2 and parkinsonism, which might have important implications for future research and treatments.

## Introduction

Infectious causes for movement disorders are not a rare occurrence and are among the most common causes of secondary movement disorders, with dystonia and parkinsonism being the most prevalent forms (Netravathi et al. 2012). While the former tends to be the dominant type of secondary movement disorder observed in children, the latter becomes more prevalent in adults, suggesting that age at insult influences the clinical picture (Netravathi et al. 2012). The relationship between viruses and parkinsonism has been recently brought under the spotlight due to the ongoing Coronavirus Disease 2019 (COVID-19) pandemic (Ghosh et al. 2021a). The reason behind such interest has historical roots in the observations relating to the ‘encephalitis lethargica’ and post-encephalic parkinsonism, which was linked to the Influenza A virus subtype H1N1 pandemic which occurred over a century ago (Taubenberger 2006; Xing et al. 2022). In addition, exposure to other viruses is known to be associated with an increased risk for the development of Parkinson’s disease (PD); these include influenza, herpes simplex, and hepatitis B (HBV) and C virus (HCV) as shown in a recent meta-analysis (Wang et al. 2020). In this review, we summarise the available evidence on viral-induced parkinsonism with a focus on postulated pathophysiological mechanisms and clinical features. We also review the evidence on viral infections and risk of developing PD.

## Viruses and parkinsonism

**Fig. 1:**
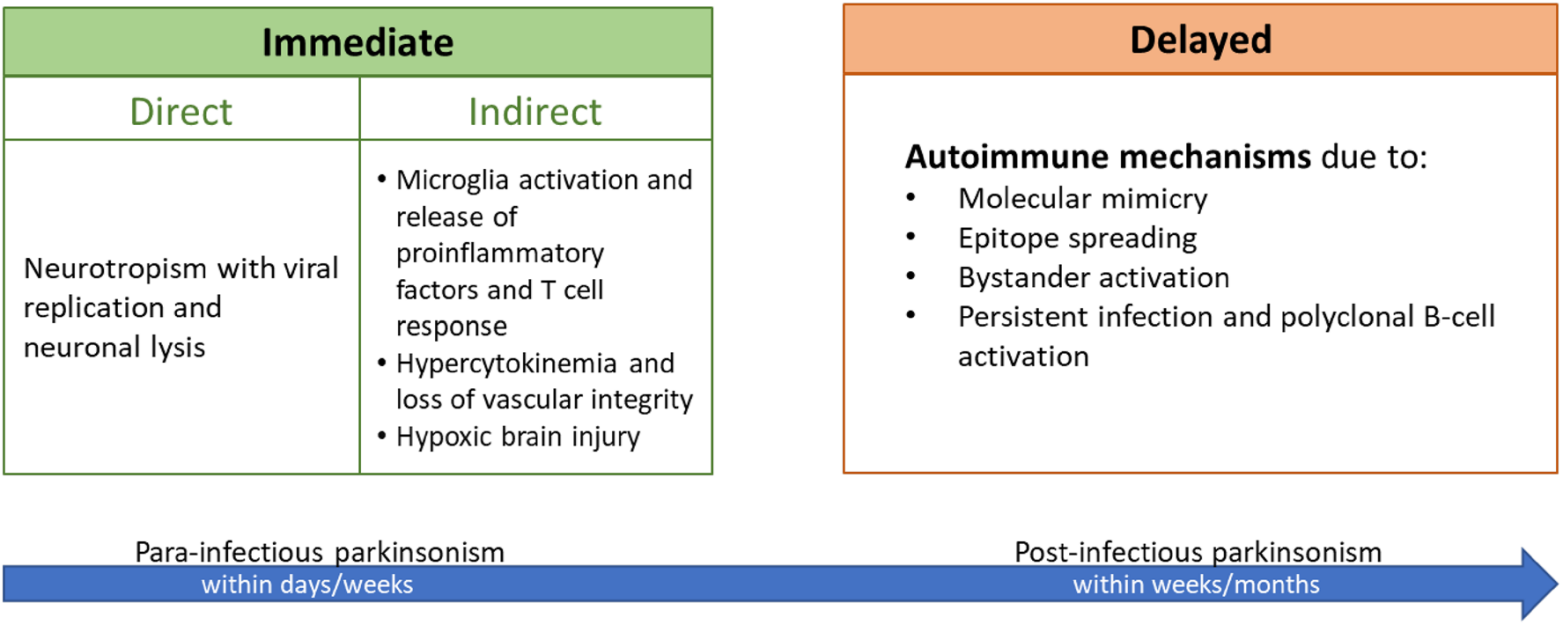
Putative pathophysiological mechanisms underlying viral infection-induced parkinsonism

Viral infections can induce parkinsonism by immediate or delayed mechanisms of damage resulting in para-infectious and post-infectious forms of parkinsonism, respectively (Fig. 1 and Table 1). Of note, the two types of damage can often coexist (Sulzer et al. 2020).

**Table 1:** Viruses and parkinsonism: agents, clinical features and management clues

Virus	Time	Transmission	Pathogenesis	Phenomenology	Diagnostic Workup	Treatment	Prognosis
*HIV*	Months to years	Sexual contact, blood, or from mother to infant	Neuroinvasion; Opportunistic infections; Drug-induced; PML-related (rare)	Atypical presentation: lack of rest tremor, PIGD phenotype, poor levodopa response	MRI: cortical atrophy and basal ganglia lesion	Poor levodopa response; HAART may be helpful in the control of parkinsonism	Usually poor prognosis
*West Nile Virus*^a^	2 to 14 days	Zoonosis (Mosquitos, Culex species)	Neuroinvasion	Typical presentation	MRI: bilateral basal ganglia, thalamus or pontine lesion; IgM in CSF or serum	Usually good control with levodopa	Usually self-limiting
*Japanese Encephalitis virus*^a^	2 to 6 weeks after the acute phase	Zoonosis (Mosquitos, Culex species)	Neuroinvasion	Atypical parkinsonism: prominent hypomimia and hypophonia	MRI: typical bilateral thalamic involvement or substantia nigra lesion	Usually good control with levodopa	Usually self-limiting
*Influenza virus*	2 weeks	Droplet, aerosol and contact (respiratory route)	Neuroinvasion	Typical presentation	–	Mild improvement with levodopa	Variable
*EBV*	2 weeks	Primarily saliva; other body fluids	Neuroinvasion, Cross-reactivity between antigens	Atypical parkinsonism: akinetic-rigid mutism, apraxia of eyelid opening, pyramidal signs	MRI: striatal hyperintensity, basal ganglia necrosis	Poor levodopa response	Variable

^a^West Nile Virus and Japanese Encephalitis virus are arboviruses (arthropod-borne viruses); transmission occurs through bite by an arthropod vector (mosquito, Culex species)

*HIV* human immunodeficiency virus, *PML* progressive multifocal leukoencephalopathy, *PIGD* postural instability and gait disorders, *MRI* magnetic resonance imaging, *HAART* highly active antiretroviral therapy,* IgM* immunoglobulin M,* CSF *Cerebrospinal fluid,* EBV* Epstein–Barr virus

The term “para-infectious” indicates a clinical event that occurs within 15 days from the infectious episode. A para-infectious parkinsonism might be underpinned by direct or indirect pathophysiological mechanisms (Bozzola et al. 2021). Viruses can be neurotropic and directly damage the nigrostriatal pathway accessing the central nervous system (CNS) via three possible routes of entry: 1. peripheral nerves; 2. blood–brain barrier (BBB); 3. blood-cerebrospinal fluid barrier (Fig. 1). Moreover, a viral infection can indirectly damage the nigrostriatal pathway via inducing inflammatory, vascular and/or hypoxic injury (Fig. 1).

Post-infectious parkinsonisms are thought to occur through a pathogen-induced autoimmunity. This may be the result of a non-specific immune activation or due to a targeted immune response to a specific host antigen. Furthermore, persistent infections may continue to drive immune responses leading to chronic inflammation or development of autoimmune processes, resulting in chronic immune stimulations that drive immune-mediated neurologic complications and damage to the nervous system (Johnson and Nath 2018). Of note, viruses can trigger autoimmune reactions through multiple mechanisms including molecular mimicry, bystander activation, and viral persistence with or without epitope spreading (Fig. 1). Molecular mimicry occurs when structural similarities between viral and host antigens trigger activation of T or B cell responses targeting both host and auto-antigens (Blackburn and Wang 2020), for example in the case of Herpes simplex virus 1 (HSV-1) and Epstein-Barr virus (EBV) (Caggiu et al. 2016, 2017; Woulfe et al. 2000). In addition, virus-specific T cells can initiate a bystander activation. Once the virus-specific T cells encounter virus-infected cells, the latter present viral peptides in the context of MHC human leukocyte antigen (HLA) class I or class II molecules to virus-specific CD8 + or CD4 + T cells respectively. This is followed by release of cytokines such as tumour necrosis factor (TNF), lymphotoxin (LT), and nitric oxide (NO), which can lead to bystander killing of the uninfected neighbouring cells (Fujinami et al. 2006). Epitope spreading is a phenomenon related to the bystander activation, which further propagates the inflammatory response. The initial immune response to an antigen is limited to particular peptide sequences on the targeted protein. Subsequently, different epitopes on the inciting antigen, or wholly distinct antigens are involved in the response, which is described as epitope spreading (Blackburn and Wang 2020). Persistent infection, in turn, leads to polyclonal proliferation of B and T cells thereby contributing to autoimmunity (Blackburn and Wang 2020). Virus-specific mechanisms of entry into the CNS and damage are described in the following sections.

### Human immunodeficiency virus (HIV)

Human immunodeficiency virus (HIV) is a retrovirus that has been shown to be the cause of acquired immunodeficiency syndrome (AIDS) (Gallo et al. 1984). An estimated 37.9 million individuals are living with HIV worldwide. Infection with HIV results in a failure of the immune system, leading to life-threatening opportunistic infections. The predominant cellular lesion in HIV infection is a progressive loss of CD4+ T cells whose levels correlate with the viral load (Ghosn et al. 2018). CNS involvement can occur quickly as HIV may enter the nervous system within the first weeks after the infection (Schacker et al. 1996; Pilcher et al. 2001).

The mechanisms of HIV entry into the brain include the ‘Trojan horse’ model (migration of the virus across the BBB via infected monocytes or lymphocytes) or the free virions model (through infected endothelial cells) (Ene 2018). Once in the brain, HIV has been clearly shown to infect astrocytes, microglia (Budka 1990; Brack-Werner 1999) and neurons (Wheeler et al. 2006; Cantó-Nogués et al. 2005). It has been proposed that HIV infection of monocytes and macrophages leads to the production of neurotoxins. These putative toxins include HIV proteins like gp120 and tat, cytokines, NO, glutamate, and quinolinic acid  (Koutsilieri et al. 2002; Lopez et al. 1999; Sardar et al. 1996). HIV proteins gp120 and tat have been linked with neuronal injury in a series of experiments (Lipton 1992). Subcortical involvement, including involvement of the basal ganglia, has been demonstrated in HIV infection. Autopsy findings by Navia et al. in patients with HIV-associated dementia showed microglial nodule encephalitis with multinucleated giant cells, particularly involving the putamen and caudate nuclei  (Navia et al. 1986). Autopsy studies in clinically asymptomatic patients by Reyes et al. showed nigral degeneration with neuronal loss, extracellular melanin and reactive astrocytosis (Reyes et al. 1991).

Patients with AIDS may develop parkinsonism as part of an HIV-related encephalopathy associated with severe damage to the dopaminergic basal ganglia (Clifford 2000). Exposure to antidopaminergic drugs can often trigger an akinetic rigid syndrome due to the state of vulnerability unleashed by the nigral degeneration due to HIV neuroinvasion (Tse et al. 2004). Rarely, parkinsonism can be the presentation of an underlying opportunistic infection (Carrazana et al. 1989). Indeed, parkinsonism has also been reported in association with progressive multifocal leukoencephalopathy (Werring and Chaudhuri 1996) and CNS tuberculosis. HIV-related parkinsonism is typically early-onset (Limphaibool et al. 2019), non–levodopa-responsive, and has often an atypical presentation with symmetrical bradykinesia and rigidity. Rest tremor is usually absent and postural tremor is prominent with early presentation of postural instability and gait difficulties. The parkinsonism observed in patients with AIDS is often associated with dementia, seizures, vacuolar myelopathy and peripheral neuropathy (Mirsattari et al. 1998; Mattos et al. 2002). Primary HIV-associated parkinsonism often appears within several months of HIV infection and is associated with a poor prognosis (Mattos et al. 2002). Diffuse nigral degeneration with loss of both pigmented and non-pigmented neurons, extracellular melanin and reactive astrocytosis have been well-documented even in clinically asymptomatic AIDS patients (Reyes et al. 1991) with a pattern of degeneration that is quite different from the predominantly ventro-lateral nigral neuronal loss seen in idiopathic PD (Itoh et al. 2000). The nigral neuroinvasive potential could explain both the occurrence of parkinsonism and the susceptibility to develop a drug-induced parkinsonism. The management of patients with HIV who present with parkinsonism involves identification and treatment of opportunistic infections, careful review of the patient’s medications for potential extrapyramidal side effects and the use of highly active antiretroviral therapy (HAART). Symptomatic treatment of the movement disorder is often unsatisfying. Evidence suggests that HAART may be helpful in the control and prevention of parkinsonsm in patients who are HIV positive (Rosso et al. 2009).

### West Nile virus (WNV)

West Nile virus (WNV) is a mosquito-borne flavivirus and human neuropathogen that has been implicated in several outbreaks in the last two decades (Campbell et al. 2002). The incubation period usually ranges from 2 to 14 days (Zou et al. 2010). Most human infections are subclinical or manifest as a mild febrile illness, but 1% of patients may develop an acute neurologic syndrome, including meningitis, encephalitis, acute flaccid paralysis, and movement disorders (parkinsonism, tremor, opsoclonus-myoclonus, chorea, ataxia, and myoclonus) (Lenka et al. 2019). Neurological complications are more frequently observed in the elderly, alcoholics, and transplant recipients (Lenka et al. 2019).

After the mosquito bite, viral replication occurs in lymph nodes and the spleen, following which viral dissemination occurs in the bloodstream. Although the precise mechanism for WNV entry into the CNS has not been delineated, preclinical studies suggest that Toll-like receptor 3 (TLR-3) and peripheral production of TNF‐α may lead to breakdown of the BBB, thereby facilitating viral entry into the CNS (Wang et al. 2004). Samuel et al. demonstrated retrograde axonal transport through the peripheral neurons, thereby leading to CNS invasion in hamster models (Samuel et al. 2007). Several other mechanisms of CNS entry, including endothelial cell infection and migration into the brain parenchyma via WNV-infected leucocytes migrating into CNS (Sejvar 2016), have been postulated. Neuronal infection is followed by cell death. Histopathologic findings in humans  are characterized by the presence of microglial nodules composed of lymphocytes and histiocytes, while leptomeningeal mononuclear inflammatory infiltrates are present in cases of meningitis. CD8 T-lymphocytes represent the predominant inflammatory cell type in the nodules and infiltrates (Sampson et al. 2000; Doron et al. 2003).

The emergence of parkinsonism in WNV infection is likely secondary to the specific neurotropism of WNV for deep gray matter nuclei, especially the substantia nigra (Lenka et al. 2019; Bosanko et al. 2003; Solomon et al. 2003). Parkinsonian features are usually self-limiting, but tremor persists for a long time after the infection in 10% of the cases with subsequent disability (Patel et al. 2015). Brain MRI may show abnormalities in the basal ganglia, thalamus, and pons, mostly bilaterally, evident in T2 and DWI sequences (Sejvar 2014). WNV infection is most sensitively assessed by the presence of immunoglobulin M (IgM) antibody in the CSF (Solomon et al. 2003). To date, no specific treatment for WNV other than symptomatic measures are available.

### Japanese encephalitis B virus (JEV)

Japanese encephalitis virus (JEV) is a flavivirus causing a mosquite-borne disease which is endemic in Southeast Asia and representing the most common cause of encephalitis (Diagana et al. 2007). Unlike other flaviviruses, JEV leads to frequent and severe neurological complications (movement disorders, poliomyelitis-like flaccid paralysis, seizures, Guillain-Barré syndrome, etc.), often leading to severe disability, while systemic features including haemorrhage and jaundice are infrequent (Turtle and Solomon 2018). JEV affects mostly  children in rural areas. (Turtle and Solomon 2018)

JEV enters the bloodstream through the bite of Culex mosquito (Diagana et al. 2007). Subsequently, binding to the CNS vascular endothelial cells allows entry into CNS; BBB disruption ensues owing to the host inflammatory response (Hsieh et al. 2019). Kalia et al. demonstrated that the mechanism of entry of JE virions into neuronal cells involves dynamin and plasma membrane cholesterol (Kalia et al. 2013). Direct neuronal damage owing to viral replication within neuronal cells and an inflammatory response have been linked to neuronal apoptosis (Raung et al. 2001). Interleukin 6 (IL-6), TNF‐α, monocyte chemotactic protein 1 (MCP1), and Regulated upon Activation, Normal T Cell Expressed and Presumably Secreted (RANTES) are thought to mediate the inflammatory response (Ghoshal et al. 2007; Chen et al. 2004).

JEV targets dopaminergic neuron-rich areas of the brain such as thalamus and midbrain. JEV-infected Fisher rats showed profound gliosis in the substantia nigra pars compacta (SNpc), similar to that seen in PD (Ogata et al. 1997). Hamue et al. reported reduced norepinephrine and dopamine levels in rats infected by JEV (Hamaue et al. 2006). Misra et al. described lower CSF concentrations of dopamine, norepinephrine and homovanillic acid, in patients with JE-associated movement disorders, stemming from impairment of the dopaminergic and norepinephrinergic systems (Misra et al. 2005).

JEV infection begins as an undifferentiated febrile illness (Turtle and Solomon 2018). If the encephalitic syndrome occurs, the brain regions noted to become involved include the thalamus, basal ganglia, brain stem, cerebellum, hippocampus, and cerebral cortex (Ishii et al. 1977). Parkinsonism is observed in a significant proportion of patients 2–6 weeks after the acute encephalitic phase (Misra and Kalita 1997), characterized by bradykinesia, rigidity, prominent masked facies and hypophonia. JEV-related parkinsonism tends to regress in most in 1–3 weeks although some patients manifest permanent sequelae, especially hypophonia (Murgod et al. 2001; Misra and Kalita 2002). Neuroimaging in patients with JEV infection may show lesions in the substantia nigra and typical extensive bilateral thalamic involvement, with a propensity for petechial haemorrhages (Murgod et al. 2001; Kumar et al. 1997). To date, no specific treatment for Japanese encephalitis other than symptomatic measures has been shown to work (Turtle and Solomon 2018).

### Influenza and the case of Encephalitis Lethargica/post-encephalitic Parkinsonism

The causative association between parkinsonism and infections may be very intricate. While many viruses could cause a para-infectious parkinsonian syndrome for their predilection for basal ganglia and substantia nigra neurotropism, as explained in the previous sections, the pathogenic mechanisms of a post-infectious  parkinsonism, whose paradigm is post-encephalitic parkinsonism (PEP), remain elusive. PEP has been historically linked to encephalitis lethargica (EL) and, together, they have been defined as one of the biggest medical mysteries (Hoffman and Vilensky 2017). EL was a polymorphic epidemic neurological syndrome which spread across Europe and then the world in the early part of the twentieth century. Firstly described by von Economo in 1917 (Hoffman and Vilensky 2017; Vilensky et al. 2010a), EL was clinically characterized by flu-like symptoms, sleepiness, disorders of ocular motility, fever, and movement disorders (both hypo- and hyperkinetic), although virtually any neurological sign or symptom could be exhibited. PEP is considered to have occurred months to years later and was most commonly characterized by akinetic-rigid features. Interestingly, decades later some of these individuals responded clinically to levodopa (Hoffman and Vilensky 2017). In the decades following the epidemic, it was estimated that as many as 50% of parkinsonian cases were postencephalitic (Krusz et al. 1987). Neuropathology of postencephalitic parkinsonism shows diffuse brain atrophy and marked neuronal loss and gliosis particularly in the substantia nigra and, interestingly, a distribution of neurofibrillary tangles that is nearly identical histopathologically to what can be observed in other neurodegenerative parkinsonisms (Jellinger 2009). The aetiology of this clinical entity is unknown, but it is generally assumed that, considering the influenza-like prodrome of the acute phase and the contemporaneous Spanish H1N1 influenza A pandemic, EL had a viral aetiology (Vilensky et al. 2010a). In fact, the 1918 flu pandemic affected large parts of the world population and is thought to have killed at least 40 million people during the same decade (Reid et al. 2001). Although Gamboa et al. have demonstrated the presence of viral antigens in the hypothalamus and midbrain of patients with PEP (Gamboa et al. 1974), other authors failed to identify the presence of the virus (Berger and Vilensky 2014). Interestingly, it has been demonstrated that several types of influenza A virus can travel into the nervous system following a systemic infection (Tanaka et al. 2003), can localize selectively in the ventral substantia nigra and hippocampus (Takahashi et al. 1995) and can induce neuroinflammation, protein aggregation and degeneration of dopaminergic neurons in the substantia nigra pars compact (SNpc) (Jang and Dezzutti 2009). Although the dopaminergic neuronal perturbation appears to be transient, the inflammatory response with permanent activation of microglia seems to persistas a long-term effect (Jang et al. 2012). Recent studies have questioned the simplistic assertion that EL led directly to PEP (Vilensky et al. 2010a, b) and have reconsidered the causative role of an infection in the context of an autoimmune disorder that could have been triggered by an infectious agent (Dale et al. 2004).

### Other post-viral parkinsonism

A number of viruses have been associated with both acute and chronic parkinsonism. Apart from the above-mentioned viral agents, other viruses associated with parkinsonism are Coxsackie (Walters 1960; Poser et al. 1969), Western equine encephalitis (Mulder et al. 1951), EBV (Espay and Henderson 2011; Dimova et al. 2006), Cytomegalovirus (Giraldi et al. 1991), St. Louis virus (Pranzatelli et al. 1994), Herpes simplex virus (Ickenstein et al. 1999), and Poliovirus (Vincent and Myers 1978).

## Viruses and idiopathic Parkinson’s disease

Several viral infections have been associated to an increased risk of developing PD (Smeyne et al. 2020) and the observation of PD-like symptoms in individuals following viral infections (see paragraph *Viruses and parkinsonism* above) has led to the hypothesis of a contributory role of viral infections in the pathogenesis of idiopathic PD. Evidence supporting this hypothesis comes from preclinical studies looking at the potential associations between α-synuclein accumulation and viral infections, as well as from epidemiological studies reporting increased risk of developing idiopathic PD following a viral infection.

Among these, influenza viruses are among those with the most robust evidence. In a study conducted on human mesencephalic dopaminergic cells in vitro and in *Rag* knockout mice in vivo, it has been shown that acute H1N1 infection led to the formation of α-synuclein aggregates through a H1N1-induced blocking of autophagosome formation and inhibition of autophagic flux (Marreiros et al. 2020). This would suggest that an aberrant proteostasis induced by the virus may play a role in the initiation of the protein misfolding process. Of note, it has been shown that treatment with oseltamivir phosphate, an anti-influenza compound, prevented H1N1-induced α-synuclein aggregation, and vaccination or treatment with oseltamivir carboxylate improved the dopaminergic neuron integrity in a model of 1-methyl-4-phenyl-1,2,3,6-tetrahydropyridine (MPTP)-treated animals infected with H1N1 (Marreiros et al. 2020; Sadasivan et al. 2017). An earlier study conducted on a mouse model infected by the H5N1 avian influenza virus showed that the virus could travel from the peripheral into the CNS, causing activation of microglia together with α-synuclein phosphorylation and aggregation in the infected regions, which persisted long after the resolution of the infection (Jang et al. 2009). In addition, a significant loss of dopaminergic neurons in the SNpc was found 60 days after the infection (Jang et al. 2009). Epidemiological studies have provided support to the possible pathogenic role of influenza viruses in idiopathic PD, although with some controversies. While a UK community-based study conducted in 2009 found an association between influenza diagnosis and PD-like symptoms but not development of idiopathic PD (Toovey et al. 2011), a subsequent population-based Canadian study found that severe influenza was associated with subsequent development of idiopathic PD (odds ratio: 2.01; 95% confidence interval: 1.16–3.48), although the association was less significant for infections occurred more than 10 years before PD diagnosis (Harris et al. 2012). Interestingly, in a recent Danish case–control study influenza was significantly associated with diagnoses of PD more than 10 years after infection, while other infections did not show such association, suggesting that a long-term association, although not proving causality, may strengthen the hypothesis of the role of influenza virus in contributing or triggering PD-related neurodegeneration (Cocoros et al. 2021).

Hepatitis virus infection has also been proposed as a potential pathophysiological factor in PD. HCV, in particular, has been linked with idiopathic PD in several epidemiological studies (Wu et al. 2015; Tsai et al. 2016; Pakpoor et al. 2017; Goldstein et al. 2019; Choi et al. 2020). Two meta-analyses (Wang et al. 2020; Wijarnpreecha et al. 2018) have demonstrated an increased risk of PD among HCV-infected patients. Interestingly, Su et al. (2019) found that PegIFN/RBV antiviral therapy was associated with a reduced risk of parkinsonism or PD in a cohort of patients with chronic HCV. Of note, Goldstein et al. (2019) found an increased risk for PD also in the group of patients with non-alcoholic steatohepatitis, arguing that liver disease per se could be a risk factor for PD rather than the viral infection and that a possible misclassification of cirrhosis-induced parkinsonism as PD could at least partially explain the association between HCV and PD. However, the mechanisms behind the link between HCV and PD is still unclear. In a study conducted by Wu et al. (2015) on midbrain neuron-glia coculture system in rats, HCV infection induced 60% of dopaminergic neuron death, similar to that of MPTP, suggesting the massive release of inflammatory cytokines might be the trigger for the observed PD-related pathological changes. While the neurotropism of HCV has now been established (Fletcher et al. 2012), it is not clear, however, if the neuronal damage could be caused by the viral replication at the CNS level or by the immune response of the infected cells (Amor et al. 2010). In addition, alteration of striatal dopaminergic transporter binding has been observed in HCV-infected patients with chronic fatigue and cognitive impairment, suggesting a defective dopaminergic transmission that could potentially underlie the association between HCV and PD (Weissenborn et al. 2006). Whether there is an association between HBV and PD is still uncertain, with some epidemiological studies reporting an increased risk of PD in HBV-infected individuals (Pakpoor et al. 2017; Choi et al. 2020) and others reporting inconsistent results (Wu et al. 2015; Tsai et al. 2016; Goldstein et al. 2019; Wang et al. 2020).

Studies have shown that EBV, a double-stranded DNA virus, can replicate in the CNS and disrupt the integrity of the BBB, causing neuronal damage and inflammation (Gent et al. 2014; Liu and Cohen 2016). It has been demonstrated that patients with PD are significantly more seropositive for EBV than the general population (Bu et al. 2015). Furthermore, it has been hypothesized that latent EBV infection can trigger autoantibodies targeting the critical repeat region cross-react with the homologous epitope on α-synuclein and induce its oligomerization (Woulfe et al. 2000).

While parkinsonism has been recognised as a potential manifestation of HIV-associated encephalopathy, it is still unclear whether HIV infection plays a role in the pathogenesis of idiopathic PD. A recent study by Santerre et al. (2021) investigated the effect of exposure to HIV-1 Vpr protein on human neuroblastoma cells (which share many features with dopaminergic neurons) and it was observed that HIV-1 Vpr protein triggers the accumulation of α-synuclein in neurons after decreasing lysosomal acidification, deregulating lysosome positioning, and the expression levels of several proteins involved in lysosomal maturation, suggesting that the suppression of neuronal autophagy exerted by the viral protein could be a mechanism responsible for toxic protein aggregation and subsequent neurodegeneration.

Caggiu et al. (2016) proposed a mechanism of molecular mimicry between HSV-1 and α-synuclein in membranes of dopaminergic neurons of the SNpc. The antibody response against homologous HSV peptides has been shown to be more prevalent among patients with PD thanin healthy controls. Cross-reactivity has been demonstrated between HSV-1 and human α-synuclein peptides indicating that HSV-1 may play a role in triggering an autoimmune response against the neurones of SNpc in PD. The same peptides are able to induce cell-mediated responses in PD patients highlighting the relevant role of TNF-α and neuroinflammation (Caggiu et al. 2017). Similarly, evidence for molecular mimicry between α-synuclein and viral protein of EBV has been demonstrated, highlighting this autoimmune mechanism in the pathophysiology of PD (Woulfe et al. 2000). A recent Taiwanese population-based cohort study revealed an increased risk of PD development among elderly patients with a prior diagnosis of herpes zoster when compared to those without history of herpes zoster infection (Lai et al. 2017).

## COVID-19, parkinsonism and Parkinson’s disease: present and future

PD is a chronic and progressive neurodegenerative disease warranting special consideration during the COVID-19 pandemic due to an increased vulnerability attributed to people with this condition (Lau et al. 2021). In part, this may be due to elderly and advanced-stage people with PD (PwP) developing progressive rigidity of respiratory muscles and the thoracic cavity, in addition to a weakened abnormal posture, as well as an impaired cough reflex (Wamelen et al. 2020; Emmi et al. 2022). In relation to severe acute respiratory syndrome coronavirus 2 (SARS-CoV-2) infection in PwP, pneumonia was by far the most common cause of inpatient admission, and the main cause of death (Okunoye et al. 2020), especially among older PwP with longer disease duration and those on device-aided therapies showing mortality rates of up to 40% (Antonini et al. 2020). Nonetheless, there seems to be a lack of consensus on the COVID-19 related mortality of PwP (Hippisley-Cox et al. 2021).

While acute implications of SARS-CoV-2 infection in PwP, particularly worsening of motor and non-motor symptoms, have been described in several studies (Antonini et al. 2020; Fearon and Fasano 2021), little is known about possible long term sequelae of COVID-19 in PwP. After the initial wave of COVID-19 pandemic, long-COVID or the “post–COVID-19 syndrome” emerged and is now defined by the National Institute for Health and Care Excellence in the United Kingdom as signs and symptoms that occur during or after an infection consistent with COVID-19, persisting for more than 12 weeks which are not explained by an alternative diagnosis (Sivan and Taylor 2020). According to one multi-centre cohort study, the most common long-term effects of COVID-19 in PwP are worsening of motor function, followed by increased levodopa daily dose requirements, fatigue and cognitive disturbances which includes “brain fog”. Other complications include reduced concentration and memory decline as well as sleep disturbances. However, the authors also suggested that post-COVID clinical features may be due to  an effect of quarantine and viral infection causing deterioration of the pre-existing PD features (Leta et al. 2021).

The idea of the COVID-19 pandemic unmasking parkinsonism cases in the near or distant future due to the widespread of the virus globally has recently become a source of speculations and concern to many researchers (Beauchamp et al. 2020; Brundin et al. 2020). Up to now, 20 cases of de novo parkinsonism, presenting during or shortly after a SARS-CoV-2 infection, have been described in the literature (Faber et al. 2020; Méndez-Guerrero et al. 2020; Cohen et al. 2020; Pilotto et al. 2021; Akilli and Yosunkaya 2021; Makhoul and Jankovic 2021; Roy et al. 2021; Fearon et al. 2021; Tiraboschi et al. 2021; Rass et al. 2021; Ghosh et al. 2021b; Ayele et al. 2021; Morassi et al. 2021; Cavallieri et al. 2021; Ong et al. 2022; Rao et al. 2022). None of these patients had a family history of PD (see Table 2).

**Table 2:** Characteristics of patients with new-onset post-COVID-19 parkinsonism (Boura and Chaudhuri 2022)

First author, date, Country	Age, Gender	COVID-19 severity	Onset	Encephalopathy	Authors’ diagnosis^a^	CSF	MRI	DaTscan	Response to Immuno- modulatory treatment	Response to dopaminergic drugs
Faber, 2020, Brazil	35F	Mild	10 d	–		Normal	Unremarkable	Abnormal	–	Significant
Méndez-Guerrero, 2020, Spain	58M	Severe/ ICU	38 d	** + **		Normal	Unremarkable	Abnormal	–	None
Cohen, 2020, Israel	45M	Moderate	2–3 w	–	Probable PD	Normal	Unremarkable	Abnormal	None	Significant
Pilotto, 2021, Italy	73M		0	+			Abnormal			
Akilli, 2021, Turkey	72M	Severe/ ICU	2 d	+			Unremarkable		Good	
Makhoul, 2021, USA	64F	Mild	5 d	-	Probable PD			Abnormal	–	
Roy, 2021, USA	60M	Severe/ ICU	8 d	+	Stroke		Abnormal			Significant
Fearon, 2021, Canada	46M	Severe/ ICU	38 d	+	Hypoxia		Abnormal		–	None
Tiraboschi, 2021, Italy	40F	Severe/ ICU	22 d	+		Normal	Unremarkable		Good	
Rass, 2021, Austria		Severe/ ICU	3 mo							
Ghosh, 2021, India	65F	Moderate	6 d	+	Osmotic demyelination	Normal	Abnormal		–	Significant
Ayele, 2021, Ehtiopia	35F	Severe/ ICU	1–2 w	+	Hypoxia	Normal	Abnormal			Significant
Morassi, 2021, Italy	70F	Severe	31 d	+		Normal	Unremarkable	Abnormal	Good	Modest
	73F^b^	Mild	0	+		Abnormal	Unremarkable		None	None
Cavallieri, 2021, Italy	67M	Moderate	4 mo	–	Probable PD		Unremarkable	Abnormal	–	
	45M	Mild	3 mo	–	Probable PD		Unremarkable	Abnormal	–	
Ong, 2022, Malaysia	31M	Severe	6 d	+	ANEC	abnormal	Abnormal		Good	
Rao, 2022, India	72M	Severe	14 d	–						Significant
	66M	Severe/ ICU	14 d	–		Normal	Unremarkable			Significant
	74M	Moderate	21 d	–		Normal	Unremarkable			Significant

ANEC acute necrotizing encephalopathy,* COVID-19 *Coronavirus Disease 2019,* CSF *cerebrospinal fluid,* d *day,* DaTscan *Dopamine Transporter scan,* F *female,* ICU *Intensive Care Unit,* ID *identification,* M *male;* mo *months,* MRI *magnetic resonance imaging, *PD *Parkinson’s disease,* USA *United States of America;* w *week,* y *year

^a^Authors’ suggested diagnosis, apart from possible post-infectious parkinsonism

^b^Death 1 month after discharged from hospital due to aspiration pneumonia and infected bedsores

The majority of these patients would well-fit the concept of post-infectious parkinsonism with relevant symptoms developing either in the context of encephalopathy (11 patients) or without (four patients). Immunomodulatory/immunosuppressive therapy was administered in five of the above cases with four of them exhibiting a good response and authors suggesting an underlying immune-mediated substrate. Interestingly, a case of acute necrotizing encephalopathy (ANEC) has been recognised among these latter cases. Trials of dopaminergic therapy have also been attempted in 11 of the above cases with parkinsonism in most of them responding either significantly (seven patients) or moderately (one patient), thus, suggesting an underlying, occasionally reversible, impairment of the dopaminergic pathway. More specific causes of secondary parkinsonism, precipitated by conditions often accompanying COVID-19, like respiratory impairment or a hypercoagulable state, have also been identified in some of the above occasions, including brain injury due to silent hypoxia (two patients), an acute stroke in the basal ganglia (one patient), and an extra-pontine osmotic demyelination syndrome, triggered by uncontrolled hyperglycemia (one patient). It is also of notice that four patients had a history of neuroleptic drug use prior to the emergence of parkinsonism (either chronic or in the context of COVID-19 hospitalisation to address agitation), thus, the possibility of drug-induced parkinsonism cannot be overlooked.

Considering the remaining patients, the treating physicians have suggested a diagnosis of probable PD in four of them, without, however, being able to exclude a diagnosis of post-infectious parkinsonism. One of these patients mentioned prior symptoms of prodromal PD (constipation), while two patients out of the three patients who were tested, were found positive for a heterozygous mutation in the genes of glucocerebrosidase (*GBA*) and leucine-rich repeat kinase 2 (*LRRK2).*

The temporal proximity of a new-onset parkinsonism with a COVID-19 diagnosis, along with concurrent encephalopathy in some cases, have led researchers to suspect an etiological connection between the two. Different mechanisms have been assumed to mediate these cases of supposedly para- or post-infectious parkinsonism, including structural or functional impairment of the nigrostriatal pathway, inflammatory or vascular damage, and unmasking of already active, although asymptomatic, cases of prodromal PD (Merello et al. 2021). However, with more than 5300 confirmed COVID-19 cases per 100,000 globally (as of February 2022) (Worldmeter.info 2021) and an annual incidence of about 15 PD cases per 100,000 (Tysnes and Storstein 2017), anticipating a parkinsonism wave based solely on the current 20 published cases sounds rather premature and susceptible to bias. Although a clear association between COVID-19 and a potential rise in parkinsonism cases cannot be currently justified, greater vigilance is recommended in order to timely recognise and address potential neurological manifestations of COVID-19, including parkinsonism, as the pandemic has only been 2 years in progression and long-term effects might not be evident yet.

## Conclusions

Parkinsonism secondary to viral infections is not uncommon and can be mediated by a variety of pathophysiological mechanisms leading to immediate or delayed damage of the nigrostriatal pathways. The available evidence seems to point towards a role of viral infections in the pathogenesis of PD, although this still remains a matter of debate. A clear association between SARS-Cov-2 infection and a rise in parkinsonism cases cannot be currently justified; however, greater vigilance for possible long-term neurological sequalae is encouraged and long-term follow up will provide further information on the association of SARS-CoV-2 with parkinsonism and PD.
